# Fast diffusion of silver in TiO_2_ nanotube arrays

**DOI:** 10.3762/bjnano.7.105

**Published:** 2016-08-03

**Authors:** Wanggang Zhang, Yiming Liu, Diaoyu Zhou, Hui Wang, Wei Liang, Fuqian Yang

**Affiliations:** 1 College of Materials Science and Engineering, Taiyuan University of Technology, Taiyuan Shanxi 030024, China; 2 Key Laboratory of Interface Science and Engineering in Advanced Materials, Taiyuan University of Technology, Ministry of Education, Taiyuan Shanxi 030024, China; 3 Department of Chemical and Materials Engineering, University of Kentucky, Lexington, KY 40506, USA

**Keywords:** activation energy, fast diffusion, magnetron sputtering, silver, TiO_2_ nanotube

## Abstract

Using magnetron sputtering and heat treatment, Ag@TiO_2_ nanotubes are prepared. The effects of heat-treatment temperature and heating time on the evolution of Ag nanofilms on the surface of TiO_2_ nanotubes and microstructure of Ag nanofilms are investigated by X-ray diffraction, field emission scanning electron microscopy, and transmission electron microscopy. Ag atoms migrate mainly on the outmost surface of the TiO_2_ nanotubes, and fast diffusion of Ag atoms is observed. The diffusivity for the diffusion of Ag atoms on the outmost surface of the TiO_2_ nanotubes at 400 °C is 6.87 × 10^−18^ m^2^/s, which is three orders of magnitude larger than the diffusivities for the diffusion of Ag through amorphous TiO_2_ films. The activation energy for the diffusion of Ag atoms on the outmost surface of the TiO_2_ nanotubes in the temperature range of 300 to 500 °C is 157 kJ/mol, which is less than that for the lattice diffusion of Ag and larger than that for the grain boundary diffusion. The diffusion of Ag atoms leads to the formation of Ag nanocrystals on the outmost surface of TiO_2_ nanotubes. Probably there are hardly any Ag nanocrystals formed inside the TiO_2_ nanotubes through the migration of Ag.

## Introduction

Titanium dioxide (TiO_2_) has gained great attention for various applications, such as energy storage [1–8] and photo-assisted reactions [9–14], due to its inherent semiconducting characteristics, chemical stability, high photo-conversion efficiency, non-toxicity and low cost [15–16]. TiO_2_ of low-dimensional structures, including nanotubes, nanoparticles, nanorods, mesoporous spheres, nanosheets, nanowires have been synthesized via anodic oxidation, template, and hydrothermal processing to increase the ratio of surface area to volume for the maximization of effective surface area [17]. Among the low-dimensional structures, TiO_2_ nanotubes (TNT) seem to be an ideal candidate for the applications in energy storage and photovoltaics.

The intrinsic poor electric conductivity and large bandgaps (approx. 3.4 eV for anatase TiO_2_ [18] and approx. 3.0 eV for rutile TiO_2_ [19–20]) have limited the applications of TiO_2_ of low-dimensional structures. To increase the electric performance of TiO_2_, TiO_2_-based materials have been developed by incorporating metal nanoparticles in TiO_2_ nanotube arrays, using electrochemical deposition [21], irradiation of microwave [22], reduction [23], and sol–gel process [24], which involve the use of aqueous solutions. In addition, the technique of magnetron sputtering has been used to deposit Ag nanostructures on the surface of TiO_2_ nanotube arrays. It is worth mentioning that Enachi et al. [25] heat-treated the TiO_2_ nanotube arrays after the deposition of Ag film of 50 nm on the top surface of TiO_2_ nanotube arrays and observed the formation of Ag nanodots on the top surface of the TiO_2_ nanotube arrays. However, they did not examine and discuss whether there exist Ag nanodots inside the TiO_2_ nanotubes or on the surface of the TiO_2_ nanotubes.

The microstructures of TiO_2_-based materials depend on the migration of atoms (dopants), which determine the functionality of TiO_2_-based materials. Although recent success in improving the photocatalytic activity and energy storage of Li ions in TiO_2_-based materials has been reported [26–29], the understanding of the migration of atoms (dopants) in TiO_2_ nanotube arrays, which determines the evolution of the microstructures of the TiO_2_-based materials, remains elusive. In this work, we report the discovery of the fast diffusion of silver through TiO_2_ nanotube arrays in forming Ag@TiO_2_ nanotube arrays. The Ag@TiO_2_ nanotube arrays are prepared by the heat treatment of TiO_2_ nanotube arrays coated with Ag nanofilms. The microstructures of the Ag@TiO_2_ nanotube arrays are characterized to determine the diffusivity and activation energy of the Ag diffusion on the surface of the TiO_2_ nanotubes.

## Results

**Figure 1:**
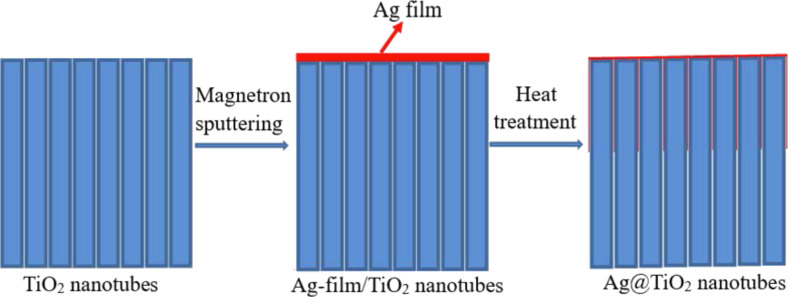
Schematic of the facile route for the preparation of Ag@TiO_2_ nanotubes.

The preparation route of the Ag@TiO_2_ nanotubes is schematically illustrated in Figure 1. The pure TiO_2_ nanotubes are prepared by a simple two-step anodization process, and then a layer of Ag film is deposited on the top of the TiO_2_ nanotubes via sputtering magnetron. The heat treatment of the TiO_2_ nanotubes with Ag nanofilm leads to the formation of Ag@TiO_2_ nanotubes.

**Figure 2:**
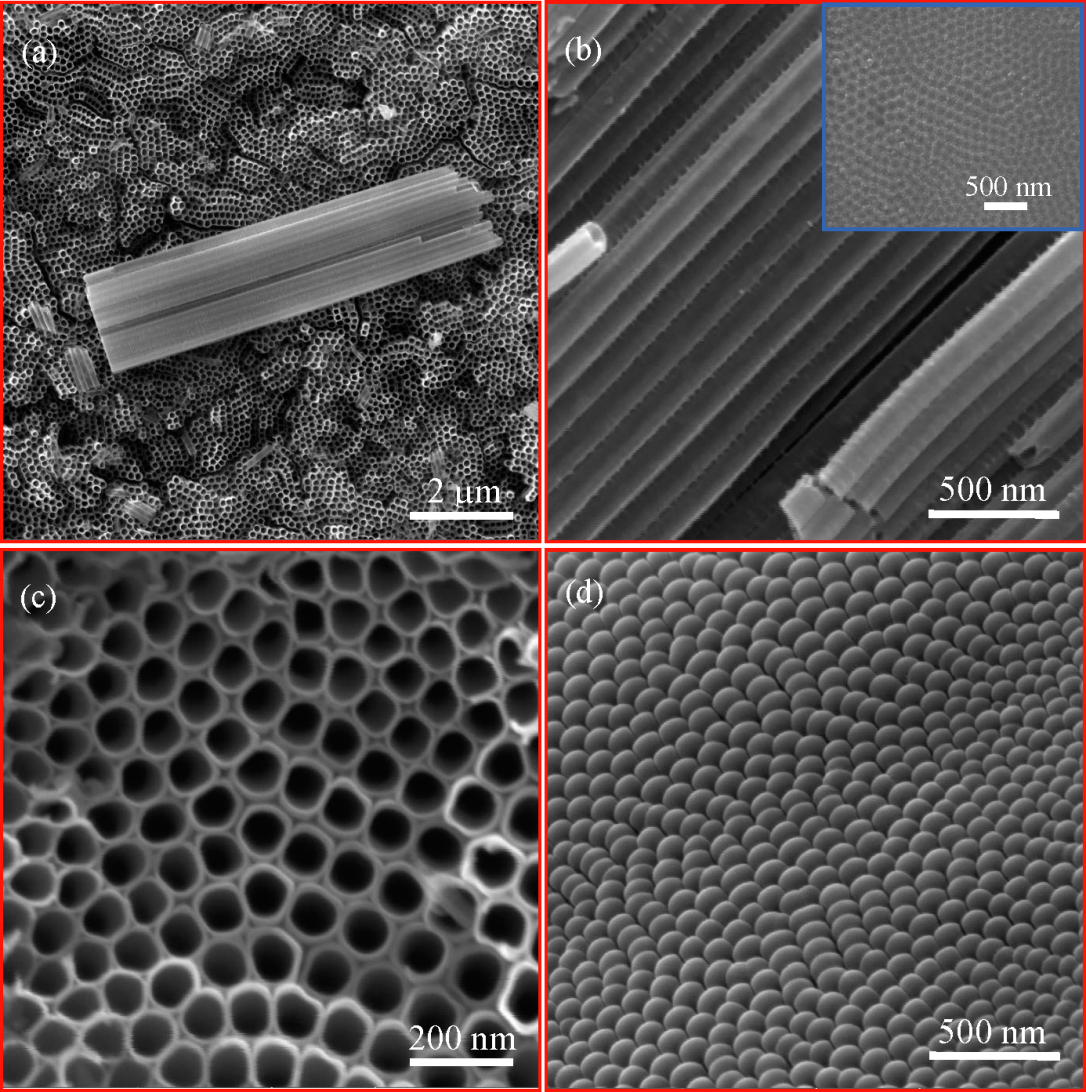
SEM images of the prepared pure TiO_2_ nanotubes by a two-step anodization process without heat treatment; (a) overview of the TiO_2_ nanotubes with an inserted image showing the average length of about 6.5 µm of the TiO_2_ nanotubes, (b) side view of the TiO_2_ nanotubes with an inserted image showing well-ordered round imprints on the Ti foil after removing the TiO_2_ nanotubes formed by a one-step anodization process, and (c) top view of the TiO_2_ nanotubes, and (d) bottom view of the TiO_2_ nanotubes.

Figure 2 shows typical SEM images of the pure TiO_2_ nanotube arrays prepared by a two-step anodization process without heat treatment. It is evident that the prepared pure TiO_2_ nanotube arrays consist of regular tubes with a diameter of 75 ± 5 nm, a wall thickness of 7 ± 2 nm and an average length of about 6.5 μm. The outmost surface of the pure TiO_2_ nanotubes prepared by the two-step anodization process is relatively clean and smooth, as shown in Figure 2b. For comparison, typical SEM images of the pure TiO_2_ nanotube arrays prepared by a one-step anodization process are shown in Figure S1c in Supporting Information File 1. The outmost surface of the pure TiO_2_ nanotubes prepared by the two-step anodization process is much cleaner and smoother than those prepared by the one-step anodization process. No “bamboo-like” structures are present on the outmost surface of the pure TiO_2_ nanotubes. Only the pure TiO_2_ nanotubes prepared by the two-step anodization process were used in this work. The inserted image in Figure 2b shows the topology of the Ti surface after the nanotubes formed by the one-step anodization were ultrasonically removed. There are no TiO_2_ nanotubes observable, and well-ordered round imprints are observed on the surface of the Ti foil.

The SEM image of Figure 2d reveals the closed honeycomb-like structure of the bottom surface of the pure TiO_2_ nanotube arrays, similar to the structure prepared by the one-step anodization process (see Figure S1d in Supporting Information File 1). The prepared pure TiO_2_ nanotube arrays exhibit a highly ordered structure, and the TiO_2_ nanotubes are normal to the surface of the corresponding Ti foil.

**Figure 3:**
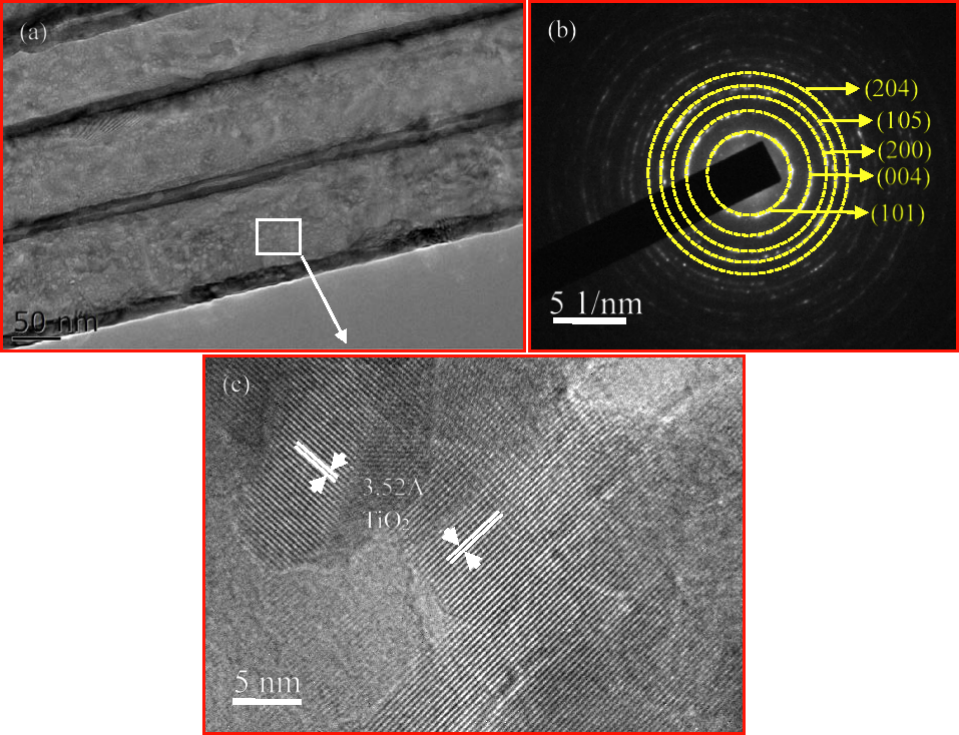
TEM images of the pure TiO_2_ nanotubes heat-treated at 500 °C for 2 h; (a) TEM image of the pure TiO_2_ nanotubes, (b) SAED pattern showing the presence of TiO_2_ nanocrystals, and (c) HRTEM image of a pure TiO_2_ nanotube.

Transmission electron microscopy (TEM) was used to analyze the microstructure of the prepared pure TiO_2_ nanotubes. Figure 3a shows the TEM images of the pure TiO_2_ nanotubes, which were heat-treated at 500 °C for 2 h. The wall thickness of the TiO_2_ nanotubes is relatively uniform along the length of the nanotubes. There is no significant change in the morphology of the pure TiO_2_ nanotubes after the heat treatment. The average inner diameter and wall thickness of the pure TiO_2_ nanotubes are ca. 75 and ca. 7 nm, respectively, in accord with the SEM observation.

The selective area electron diffraction (SAED) pattern of the pure TiO_2_ nanotubes shown in Figure 3b depicts diffusion rings, suggesting the formation of TiO_2_ polycrystals after heat treatment. From the HRTEM (high-resolution TEM) analysis shown in Figure 3c, a width of 3.52 Å between neighboring lattice fringes is observed in agreement with the (101) lattice spacing of anatase TiO_2_ [30–31]. This result reveals the highly crystalline nature of the TiO_2_ nanotubes in accord with the SAED pattern. The heat treatment can lead to a transition of TiO_2_ nanotubes from the amorphous phase to a crystalline phase, which has been observed by Jarose et al. [32].

It must be emphasized that the heat treatment with a temperature less than and equal to 500 °C does not cause any morphological changes of the pure TiO_2_ nanotubes, as demonstrated in Figure 2 and Figure 3. The outmost surface of the pure TiO_2_ nanotubes remained relatively clean and smooth.

**Figure 4:**
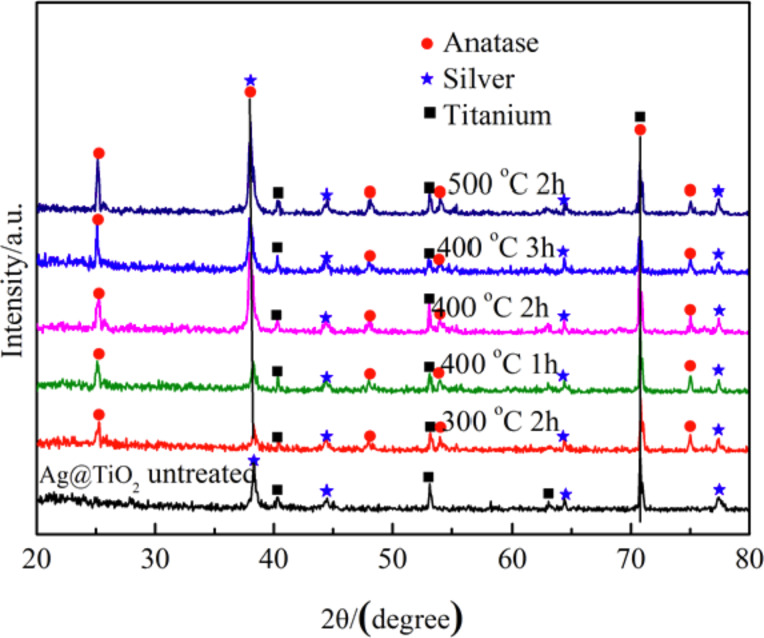
XRD patterns of TiO_2_ nanotube arrays with Ag nanofilm heat treated at three different temperatures for different periods of time.

A Ag nanofilm of 230 ± 10 nm in thickness was deposited on top of the prepared pure TiO_2_ nanotube arrays. The pure TiO_2_ nanotube arrays with Ag nanofilm were heat-treated at three temperatures of 300, 400, and 500 °C in air for different periods of time. X-ray diffraction (XRD) was used to analyze the crystal structure of Ti and Ag in the heat-treated TiO_2_ nanotube arrays with Ag nanofilm. Figure 4 shows the XRD pattern of the TiO_2_ nanotube arrays with Ag nanofilm. No crystallinity of TiO_2_ is observable for the untreated TiO_2_ nanotube arrays with Ag nanofilm, and the coating of the Ag nanofilm does not cause any change of the microstructure of the TiO_2_ nanotube arrays. The XRD patterns of all the heat-treated TiO_2_ nanotube arrays with Ag nanofilm clearly display the peaks of TiO_2_, suggesting the transition of TiO_2_ from the amorphous phase to the crystalline phase in accord with the results observed from the TEM analysis. The diffraction peaks with the 2θ values of ca. 25.3°, ca. 37.8°, ca. 48.0°, ca. 54.0° and ca. 75.0° shown in Figure 4 correspond to the spacings of the (101), (004), (200), (105), and (215) crystal lattice planes of the anatase phase of TiO_2_ in accord with the values in the standard card (JCPDS NO.21-1272). No diffraction peaks for the rutile phase of TiO_2_ are detectable. The diffraction peaks with the 2θ values of ca. 38.1°, ca. 44.3°, ca. 64.4° and ca. 77.4° correspond to spacings of the (111), (200), (220) and (311) crystal lattice planes of cubic Ag with a lattice constant of 4.0861 Å (JCPDS NO.65-2871). The diffraction peaks with the 2θ values of ca. 40.2°, ca. 53.0°, ca. 63.0° and ca. 70.7° correspond to the spacings of the (101), (102), (110) and (103) crystal lattice planes of Ti substrate (JCPDS NO.65-6231).

**Figure 5:**
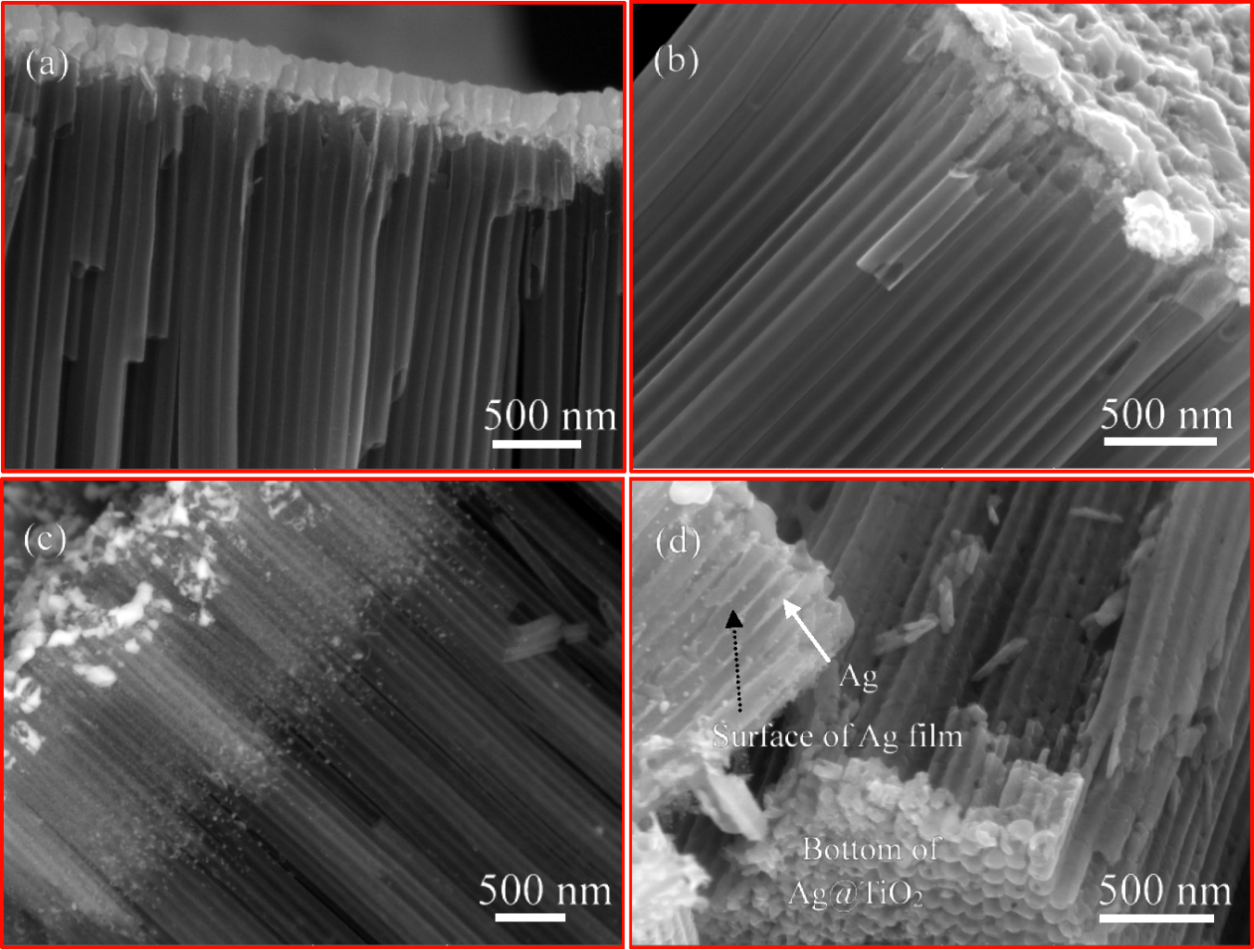
SEM images of the TiO_2_ nanotube arrays with Ag nanofilm after different heat treatment for 2 h; (a) without heat treatment, (b) heat treatment at 300 °C, (c) heat treatment at 400 °C, and (d) heat treatment at 500 °C.

Figure 5 shows SEM images of the TiO_2_ nanotube arrays with Ag nanofilm after heat treatment at different temperatures for 2 h. It is evident that a Ag nanofilm of about 225 nm was deposited on the top of the TiO_2_ nanotube arrays. Without heat treatment, there is little Ag migrating through the TiO_2_ nanotubes, as shown in Figure 5a. The outmost surface of the TiO_2_ nanotubes remains relatively clean and smooth. The magnetron sputtering did not cause any significant migration/diffusion of Ag through the TiO_2_ nanotube arrays. The Ag nanofilm generally covered the top of the TiO_2_ nanotube arrays (see Figure S2a in Supporting Information File 1) and made the topology of the TiO_2_ nanotubes indistinguishable. With the heat treatment at 300 °C for 2 h, Ag migrated slightly into the TiO_2_ nanotube arrays, as shown in Figure 5b. There are Ag nanoparticles present on the outmost surface of the TiO_2_ nanotubes. The Ag nanofilm became irregular due to the migration of Ag through the TiO_2_ nanotube arrays and dewetting of the Ag nanofilm, and the topology of TiO_2_ nanotubes become visible (see Figure S2b in Supporting Information File 1). The increase of the temperature to 400 °C for the heat treatment caused a significant amount of Ag to migrate through the TiO_2_ nanotube arrays, as shown in Figure 5c, leading to the formation of Ag nanoparticles. There was a layer of Ag over the TiO_2_ nanotubes. Ag nanoparticles scattered around the propagation front, likely representing the nucleation and growth of Ag nanoparticles induced by the migration/diffusion of Ag during the heat treatment. For the segment of the TiO_2_ nanotubes not covered by the Ag layer and Ag nanoparticles, the outmost surface remained relatively clean and smooth. Figure S3 in Supporting Information File 1 shows the line scan of EDX of the cross section of the corresponding TiO_2_ nanotube arrays with Ag nanofilm, which supports the SEM image shown in Figure 5c. Further increase of the temperature to 500 °C for the heat treatment introduced an interesting phenomenon, as shown in Figure 5d. After heat treatment at 500 °C for 2 h, Ag migrated completely through the TiO_2_ nanotube arrays to the interface between the TiO_2_ nanotubes and the Ti foil and formed a “film” of Ag covering the outmost surface of the TiO_2_ nanotubes. The Ag “film” basically occupied the space between TiO_2_ nanotubes. Ag atoms migrated through the space between TiO_2_ nanotubes instead of migrating/diffusing through the inner surface of the TiO_2_ nanotubes. There are probably hardly any Ag nanoparticles formed inside the TiO_2_ nanotubes through the migration of Ag, where some Ag nanoparticles are present on the surface of the Ag film. As shown in Figure S2 in Supporting Information File 1, more TiO_2_ nanotubes become visible for the heat treatment at higher temperatures, which confirms the migration of Ag through the TiO_2_ nanotube arrays. The increase in the temperature for the heat treatment of the TiO_2_ nanotube arrays with Ag nanofilm enhances the migration of Ag atoms, suggesting that the migration of Ag atoms is a thermal activation process.

**Figure 6:**
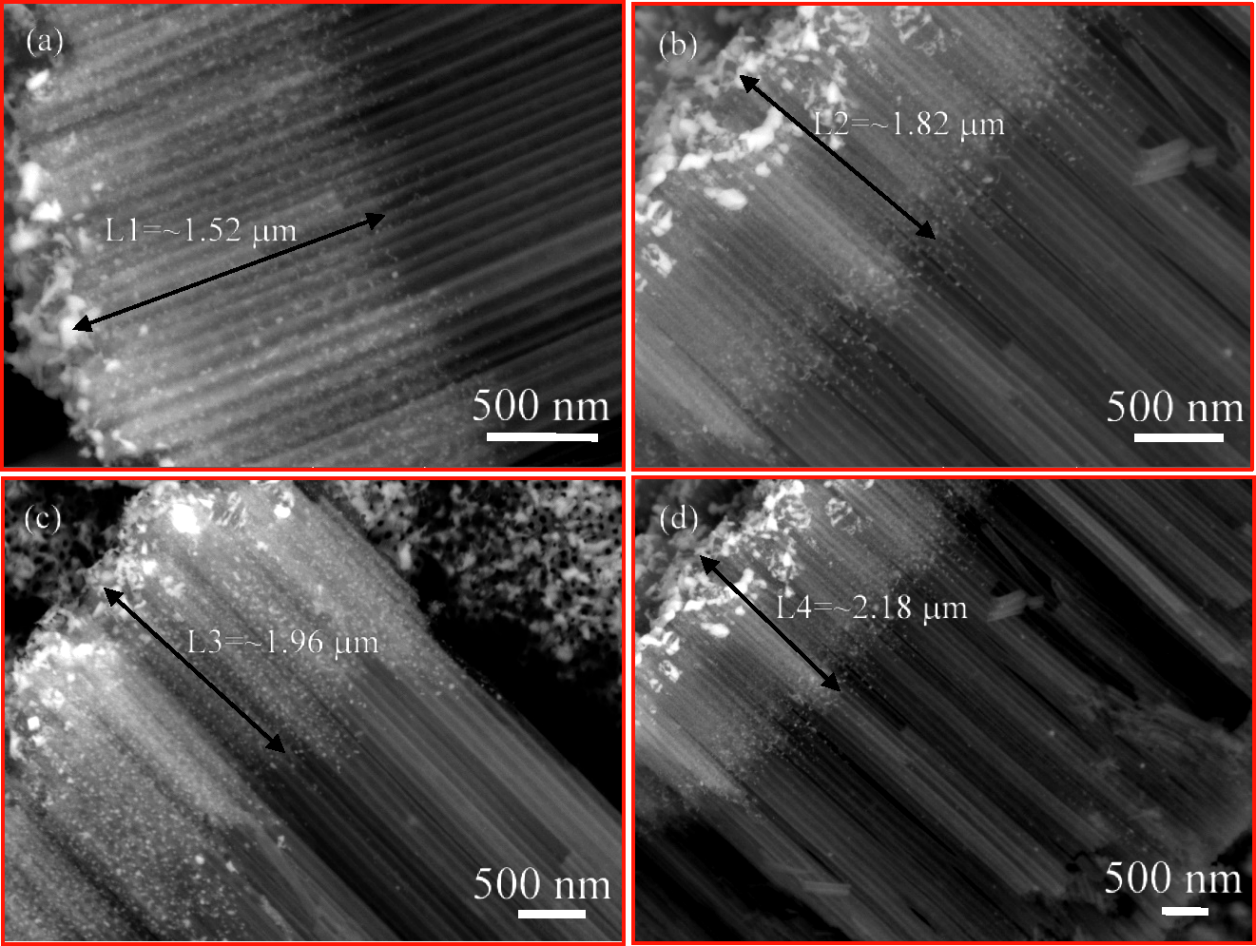
SEM images of the heat-treated TiO_2_ nanotube arrays with Ag nanofilm at 400 °C for different periods of time: (a) 1 h, (b) 2 h, (c) 3 h, and (d) 4 h.

Figure 6 shows SEM images of the TiO_2_ nanotube arrays with Ag nanofilm, which were heat treated at 400 °C for 1, 2, 3 and 4 h. It is evident that the migration/diffusion length of Ag atoms into the TiO_2_ nanotube arrays increases with the increase of the heating time, as expected. Relatively uniform migration/diffusion lengths are observed for each individual heating time, even though there are some regions with larger migration/diffusion lengths. A significant amount of Ag nanoparticles near the propagation fronts was formed after all four periods of time.

**Figure 7:**
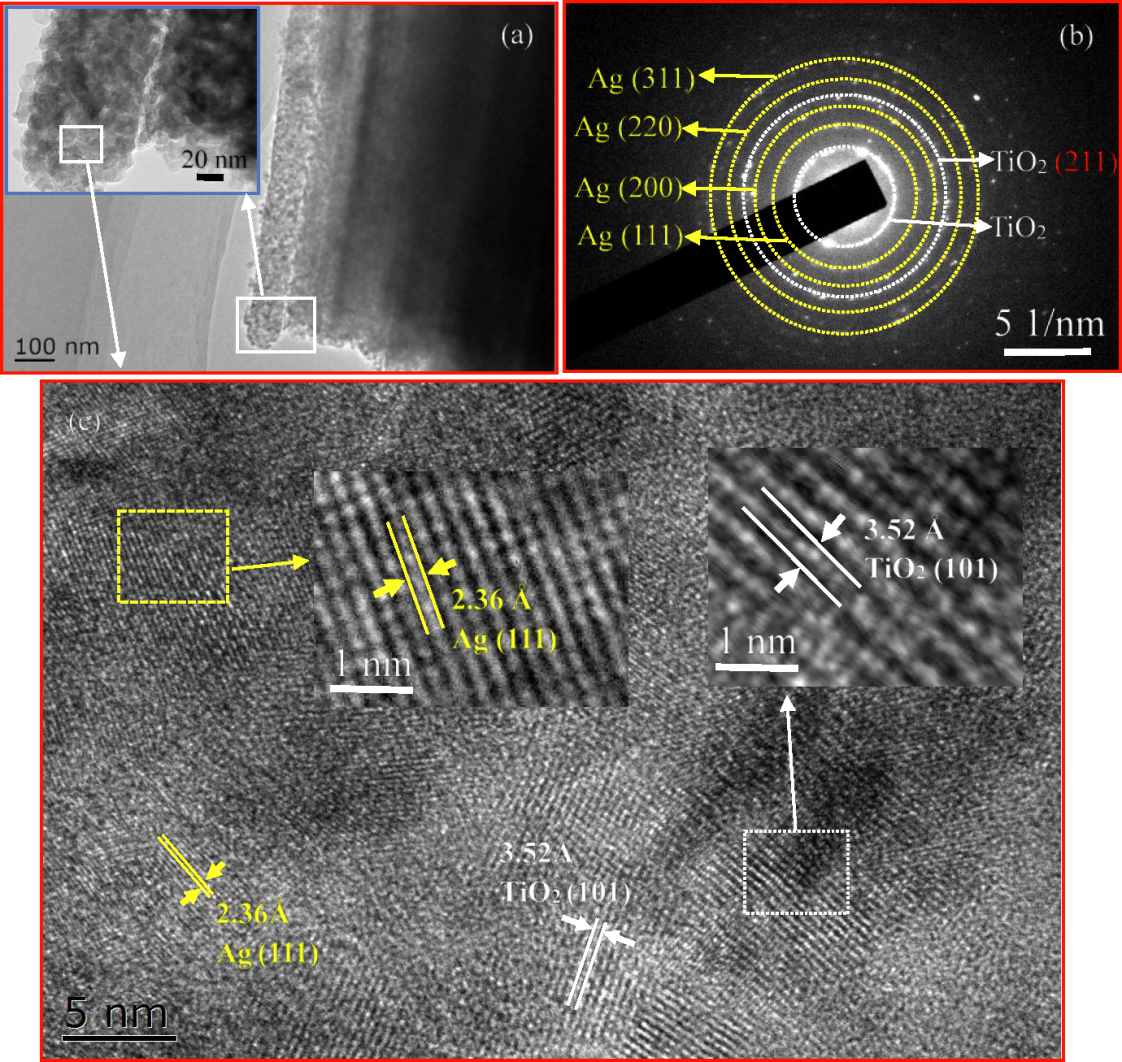
TEM images of the TiO_2_ nanotube arrays with Ag nanofilm heat treated at 500 °C for 2 h; (a) TEM image of a TiO_2_ nanotube covered by a Ag nanofilm, which consists of numerous Ag nanoparticles, (b) SAED pattern showing the presence of Ag nanocrystals, and (c) HRTEM image of the Ag nanofilm showing characteristic lattice spacings of 3.52 Å for TiO_2_ nanotubes and 2.36 Å for Ag.

TEM was used to analyze the microstructures of the heat-treated TiO_2_ nanotube arrays with Ag nanofilm. Figure 7 shows the TEM images of the TiO_2_ nanotube arrays with Ag nanofilm heat-treated at 500 °C for 2 h. The morphology of TiO_2_ nanotubes remained the same except the presence of a layer of Ag nanoparticles, as shown in Figure 7a, which is in accord with the images shown in Figure 5 and Figure 6. The migration/diffusion of Ag and heat treatment do not change the nano-tubular structure of TiO_2_. It is interesting to note that the Ag film, which covers the outmost surface of the TiO_2_ nanotubes, is not continuous and made of Ag nanoparticles of about 10 nm in size, as shown in the image embedded in Figure 7a.

The EDS result (see Figure S4 in Supporting Information File 1) reveals the presence of Ti, O, and Ag, confirming the existence of Ag around the TiO_2_ nanotubes. The SAED pattern of the Ag film on the outmost surface of TiO_2_ nanotubes, as shown in Figure 7b, displays diffuse rings, suggesting that Ag nanoparticles are present in the form of polycrystals. This result suggests that Ag was not oxidized during the heat treatment. The (111), (200), (220) and (311) crystal planes of cubic Ag are observed, in good accord with the XRD analysis. The HRTEM image shown in Figure 7c depicts the characteristic lattice fringe of 3.52 Å for TiO_2_ nanotubes and 2.36 Å for Ag, which correspond to the (101) plane of anatase TiO_2_ and {111} planes of face-centered cubic (fcc) structure of Ag [33], respectively. The Ag nanoparticles are Ag nanocrystals. There is no oxidation of Ag nanocrystals in contrast to the general statement that Ag nanoparticles are very reactive and are easily oxidized under the condition of UV irradiation, ambient atmosphere and room temperature [34]. Heat treatment at relatively high temperatures likely leads to the decomposition of silver oxide [34].

It is worth mentioning that the heat-treatment-induced migration/diffusion of Ag into the TiO_2_ nanotube arrays of anatase phase has also been observed. The SEM images in Figure S6 in Supporting Information File 1 for the TiO_2_ nanotube arrays of anatase phase with Ag film heat treated at 500 °C in air for 2 h demonstrates the presence of Ag nanoparticles on the outmost surface of the TiO_2_ nanotube arrays of anatase phase. Ag atoms can migrate into TiO_2_ nanotube arrays of either amorphous phase or anatase phase.

## Discussion

As shown in Figure 5 and Figure 6, there are probably only very few Ag nanoparticles formed inside the TiO_2_ nanotubes through the migration of Ag. Such behavior reveals the curvature effect on the distribution or wetting of Ag on the surface of the TiO_2_ nanotubes. According to the Gibbs–Thomson relation [35–36], the equilibrium concentration of solute atoms on a curved surface is determined by the surface energy and the mean curvature. Thus, the equilibrium concentration of Ag atoms on the inner surface of a TiO_2_ nanotube is lower than that on the planar surface of TiO_2_ of the same structure, and on the outer surface it is higher than that on the planar surface. Ag atoms are preferred to migrate on the outmost surface of the TiO_2_ nanotubes and form a layer of Ag during the heat treatment, as observed in Figure 5 and Figure 6.

According to the theory of thermodynamics [37], the melting point of a material is a function of the size of the material. The size dependence of the melting point of a material in spherical shape can be expressed as [38]

[1]
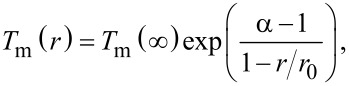


with

[2]
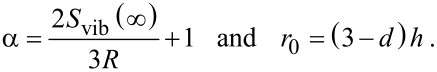


Here, *T*_m_(*r*) is the melting temperature of the material in a spherical shape of radius *r*, *T*_m_(∞) is the melting temperature of the material in bulk shape, *R* is the gas constant, *S*_vib_(∞) is the vibrational melting entropy of the material, *h* is the atomic radius of the material, and *d* = 0 for nanoparticles. As shown in Figure S7 of Supporting Information File 1, the radius of Ag nanoparticles is in the range from 2.6 ± 0.2 to 5 ± 2 nm. From Equation 1 and Equation 2, one can note that the smaller the size of nanoparticle, the lower is the melting point. Using the parameters of *h* = 0.2898 nm [39], *T*_m_(∞) = 1234 K [40], *S*_vib_(∞) = 7*.*98 J mol^−1^·K^−1^ [38] in Equation 1, one obtains a melting temperature of 857.5 K (584.5 °C) for the smallest size of Ag crystalline nanoparticles (2.4 nm), which is much higher than 300–500 °C used in the heat treatment. The formation of Ag nanocrystals on the outmost surface of the TiO_2_ nanotubes involves the nucleation and growth processes controlled by the migration/diffusion of Ag atoms.

Generally, the migration of Ag through the TiO_2_ nanotube arrays involves diffusion, which is controlled by the gradient of concentration. With the deposition of a Ag film on the top of highly ordered TiO_2_ nanotube arrays, the diffusion of Ag on the outmost surface of the TiO_2_ nanotubes can be approximated as a one-dimensional problem. The equation describing the diffusion of Ag along the outmost surface of the TiO_2_ nanotubes is

[3]
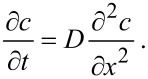


Here, *c* is an average concentration of Ag over the cross-sectional area confined by three adjacent TiO_2_ nanotubes that are in contact with each other. The condition of zero flux in the direction normal to the outmost surface was used to derive Equation 3. The concentration at the top of the TiO_2_ nanotubes can be approximated as constant, i.e.,

[4]
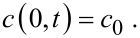


Here, *c* is the concentration of Ag atoms, *c*_0_ is the concentration of Ag atoms at the top of the TiO_2_ nanotubes, *t* is the time, *D* is the diffusivity of Ag atoms, and *x* is the migration distance of Ag on the outmost surface of the TiO_2_ nanotubes. The initial condition is

[5]
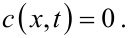


The solution of Equations 3–5 can be found as

[6]
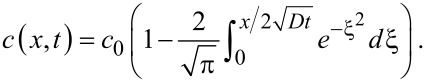


For *c*(*x*, *t*)/*c*_0_ = 0.01, one obtains

**Figure 8:**
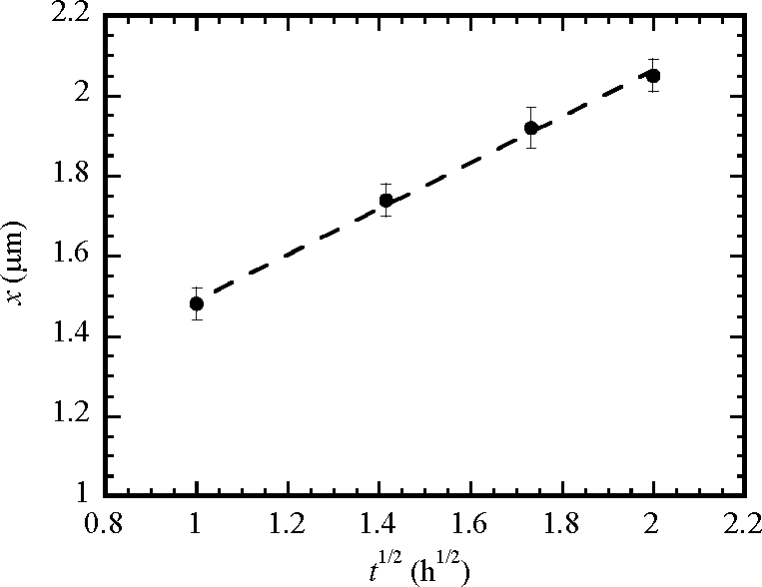
Migration distance of Ag atoms on the outmost surface of the TiO_2_ nanotubes as a function of the time during the heat treatment at 400 °C.

[7]
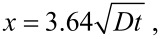


which shows that the migration distance of Ag atoms on the outmost surface of the TiO_2_ nanotubes is proportional to the square root of time, as expected for random diffusion. Using the SEM images shown in Figure 6, the temporal variation of the migration distance of Ag atoms on the outmost surface of the TiO_2_ nanotubes for the heat treatment at 400 °C is depicted in Figure 8. It is evident that there is a linear relation between the square root of the migration time and the migration distance of Ag atoms on the outmost surface of the TiO_2_ nanotubes, which is in accord with Equation 7. Using the linear regression to fit the experimental data in Figure 8, one obtains the diffusivity of 6.87 × 10^−18^ m^2^/s.

It is known that the temperature dependence of diffusivity follows an Arrhenius relation as *D* = *D*_0_·exp(−*Q*/*RT*) with *D*_0_ being a pre-exponential constant, *Q* is the activation energy for the rate process, *R* is the gas constant, and *T* is the absolute temperature. Using the Arrhenius relation, the temperature dependence of the migration distance of Ag on the outmost surface of the TiO_2_ nanotubes during the heat treatment can be expressed as

[8]
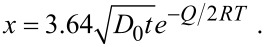


**Figure 9:**
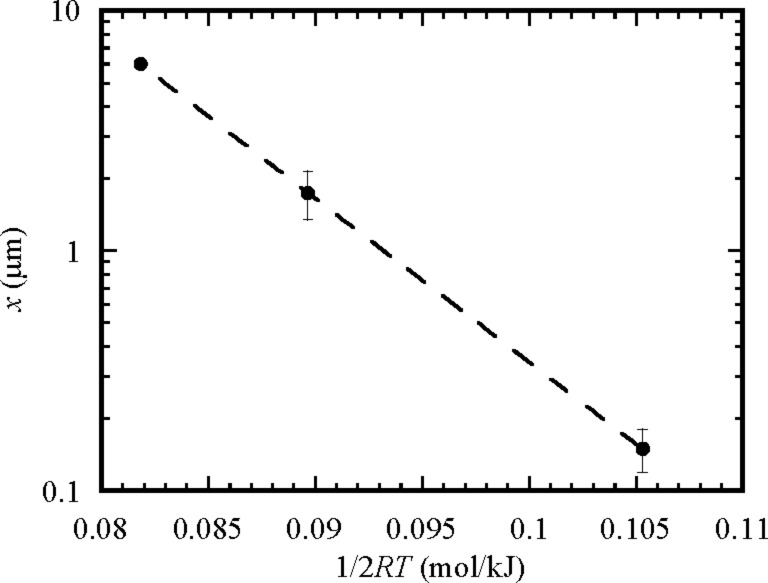
Temperature dependence of the migration distance of Ag atoms on the outmost surface of the TiO_2_ nanotubes for a heat treatment of 2 h.

As shown in Figure 5d, Ag migrated completely through the TiO_2_ nanotube arrays to the interface between the TiO_2_ nanotubes and the Ti foil during the heat treatment at 500 °C for 2 h. One can approximate the migration distance of Ag atoms on the outmost surface of the TiO_2_ nanotubes as about 6.5 μm of the length of the TiO_2_ nanotubes for the heat treatment at 500 °C for 2 h. Figure 9 shows the temperature dependence of the migration distance of Ag atoms on the outmost surface of the TiO_2_ nanotubes for a heat treatment of 2 h. Using Equation 8 to fit the experimental data shown in Figure 9, one obtains the activation energy of 157 kJ/mol for the migration/diffusion of Ag atoms on the outmost surface of the TiO_2_ nanotubes in the temperature range of 300 to 500 °C.

There are studies on the diffusion of Ag, including self-diffusion, grain boundary diffusion, and diffusion of Ag in amorphous TiO_2_ films. Table 1 summarises the data available in literature for the diffusivity and activation energy of the diffusion of Ag. For comparison, the results obtained in this work are also included in Table 1. It is evident that the diffusivity of Ag on the outmost surface of the TiO_2_ nanotubes is three orders of magnitude larger than the results reported by Kulczyk-Malecka et al. [41] for the sandwich structure of TiO_2_/Ag/TiO_2_ coatings, and the activation energy is also larger than that given by Kulczyk-Malecka et al. [41]. Such differences reveal the effect of the microstructure on the diffusion behavior of Ag. The TiO_2_ in TiO_2_/Ag/TiO_2_ coatings was present in the form of thin film and amorphous phase. Also, the time of the heat treatment for the measurement of the diffusivity was 5 min [41], which is much less than the time used in this work. It is interesting to note that the diffusivity obtained in this work is compatible with the diffusivity for the grain boundary diffusion of Ag with the activation energy being less than that for the lattice diffusion of Ag and larger than that for the grain boundary diffusion. Such a result suggests that both the grain boundary diffusion of Ag and the interface diffusion on the surface of the TiO_2_ nanotubes play important roles in controlling the migration/diffusion of Ag into the TiO_2_ nanotube arrays.

**Table 1:** Diffusivity and activation energy for the diffusion of Ag.

	temperature (°C)	diffusivity (m^2^/s)	activation energy (kJ/mol)

diffusion of Ag in TiO_2_/Ag/TiO_2_ coatings [41]	100–400	(3.5–5.8) × 10^−21^	127.5
diffusion of Ag in TiO_2_/Ag/TiO_2_ coatings [41]	600	5.93 × 10^−20^	3.5
lattice diffusion of Ag [42]	400	1.1 × 10^−19^	192.0
grain boundary diffusion of Ag [42]	400	6.0 × 10^−18^	111
Ag diffusion into Au core [43]	27	(7.5–21) × 10^−25^	
Ag on surface of TiO_2_ nanotubes (this work)	400	6.87 × 10^−18^	157

## Conclusion

In summary, an approach has been developed to coat the outmost surface of TiO_2_ nanotubes in nanotube arrays with Ag via the deposition of Ag films on top of the TiO_2_ nanotube arrays by magnetron sputtering and subsequent heat treatment at a temperature between 300 and 500 °C. A two-step anodization process was used to prepare the TiO_2_ nanotube arrays. The migration of Ag atoms through the TiO_2_ nanotube arrays was investigated ex situ by using SEM and TEM. In contrast to the Ag@TiO_2_ nanotubes prepared by the methods involving aqueous solutions, Ag nanocrystals were formed on the outmost surface of TiO_2_ nanotubes. There probably were hardly any Ag nanocrystals formed inside the TiO_2_ nanotubes through the migration of Ag. Such a result reveals that Ag atoms prefer to migrate/diffuse on the convex surface of the TiO_2_ nanotubes. The average migration/diffusion length of Ag atoms on the outmost surface of the TiO_2_ nanotubes along the longitudinal direction of the TiO_2_ nanotubes increases with the increase of the heat treatment temperature and the heating time. During the heat treatment at 500 °C for 2 h, Ag atoms migrated completely through the TiO_2_ nanotube arrays and formed an Ag nanofilm, which consists of numerous Ag nanocrystals, covering the outmost surface of the TiO_2_ nanotubes.

Using the theory of diffusion, the migration/diffusion of Ag atoms on the outmost surface of the TiO_2_ nanotubes was analyzed. The diffusivity for the diffusion of Ag atoms on the outmost surface of the TiO_2_ nanotubes at 400 °C is 6.87 × 10^−18^ m^2^/s, which is three orders of magnitude larger than the results reported by Kulczyk-Malecka et al. [41] for the sandwich structure of TiO_2_/Ag/TiO_2_ coatings. Such a result demonstrates the fast diffusion of Ag atoms on the surface of the TiO_2_ nanotubes. The activation energy for the migration/diffusion of Ag on the outmost surface of the TiO_2_ nanotubes in the temperature range of 300 to 500 °C is 157 kJ/mol, which is less than that for the lattice diffusion of Ag and larger than that for the grain boundary diffusion. Both the grain boundary diffusion of Ag and the interface diffusion on the outmost surface of the TiO_2_ nanotubes play important roles in the migration/diffusion of Ag into the TiO_2_ nanotube arrays.

## Experimental

### Preparation of TiO_2_ nanotubes

TiO_2_ nanotubes on Ti foils (99.9 atom %) were fabricated via a two-step anodization process [44]. Briefly, commercially available pure titanium foils (30 × 10 × 0.1 mm^3^, China Research Institute of Nonferrous Metals, China) were ultrasonically cleaned with acetone, ethanol, and deionized water for 15 min each. Anodization of the Ti foils was performed in a mixture of water, ethylene glycol and 0.13 M NH_4_F, using a conventional two-electrode cell system. The volume ratio of water to ethylene glycol was 2:100. The cleaned Ti foil was used as the working electrode, and a platinum foil was used as the counter electrode. The anodization voltage was 50 V, and the anodization time was 1 h. TiO_2_ nanotubes with “bamboo-like” structure at the outer layer were obtained (Figure S1c in Supporting Information File 1). The TiO_2_ nanotubes with “bamboo-like” structure were completely removed ultrasonically from the as-anodized Ti foils, resulting in well-ordered round imprints on the surface of the Ti foils (see the inserted image in Figure 2b). After being cleaned ultrasonically, the Ti foils were anodized for another 30 min under the same condition to obtain the two-step TiO_2_ nanotube arrays with relatively clean outmost surface (Figure 2b). The prepared TiO_2_ nanotube arrays were rinsed with water and dried in air.

### Deposition of Ag nanofilms on the top of TiO_2_ nanotube arrays

Ag nanofilm of 230 ± 10 nm in thickness was deposited on the top of the TiO_2_ nanotube arrays at room temperature (25 °C) via magnetron sputtering in a multifunctional magnetron sputtering instrument (JGP560B), using an Ag target. Argon gas was flowed into the sputtering chamber after the pressure of the sputtering chamber reached 8 × 10^−4^ Pa. The surface residuals were removed via pre-sputtering. During the sputtering, argon gas flowed through the chamber at a rate of 30 sccm and a chamber pressure of 6 Pa. The separation between the top of the TiO_2_ nanotubes and the target was about 30 mm.

The TiO_2_ nanotube arrays with Ag nanofilms were heat treated at temperatures of 300, 400 and 500 °C, respectively, for different holding times (1, 2 and 3 h) at a ramping rate of 2 °C·min^−1^ in a tube furnace in air. The heat treatment allowed Ag to migrate through the TiO_2_ nanotubes. Figure 1 shows schematically the facile route for the preparation of Ag@TiO_2_ nanotubes.

### Materials characterization

The morphology and selected area electron diffraction (SAED) patterns of the prepared structures were examined using field-emission scanning electron microscopy (FESEM) (Tescan MIRA3 LMH) at 10 kV and transmission electron microscopy (TEM) (JEOL 2100-F) at 200 kV. The composition of the prepared structures was determined using energy dispersive spectroscopy (EDS) (OXFORD X-Max 80), and the crystal structure of the prepared structures was analyzed with Cu Kα radiation on a Rigaku D/max 2500 X-ray diffractometer with patterns recorded in a range of 20–80°.

To prepare the TEM specimens, the as-prepared Ag@TiO_2_ nanotubes, which were scratched from the Ag@TiO_2_ nanotube arrays using a pair of sharp tweezers, were treated ultrasonically in ethanol for 5–8 min to form a suspension. A drop of the suspension was then drippled on a carbon film supported by a copper grid and dried in air before the TEM analysis.

## Supporting Information

File 1 Additional experimental results.
