# Supplementary material for: Fast diffusion of silver in TiO2 nanotube arrays
Source: Beilstein J Nanotechnol. 2016 Aug 3;7:1129–40. doi: 10.3762/bjnano.7.105 (PMC4979750; doi:10.3762/bjnano.7.105)
Supplement: File 1 — Additional experimental results. [file Beilstein_J_Nanotechnol-07-1129-s001.pdf]

Supporting Information

for

Fast diffusion of silver in TiO_2 nanotube arrays

Wanggang Zhang[1,2,3], Yiming Liu[1,2], Diaoyu Zhou[1,2], Hui Wang[1,2], Wei Liang*[1,2] and Fuqian Yang*[3]

Address: [1]College of Materials Science and Engineering, Taiyuan University of Technology, Taiyuan Shanxi 030024, China; [2] Key Laboratory of Interface Science and Engineering in Advanced Materials, Taiyuan University of Technology, Ministry of Education, Taiyuan Shanxi 030024, China and [3] Department of Chemical and Materials Engineering, University of Kentucky, Lexington, KY 40506, USA

Email: Weil Liang* - liangwei@tyut.edu.cn; Fuqian Yang* - fyang2@uky.edu

*Corresponding author

Figure S1 shows the TiO_2 nanotube arrays prepared by the one-step anodization process. The TiO_2 nanotubes exhibit a self-assembled structure. The diameter and length of the TiO_2 nanotubes are about 85 nm and about 6.5 μm, respectively, with the diameter being similar to the TiO_2 nanotubes prepared by the two-step anodization process. The SEM image of the TiO_2 nanotubes shown in Figure S1c reveals a “bamboo-like” structure on the outermost surface of the TiO_2 nanotubes. From Figure S1d, one can conclude that well-aligned nanotubes grew vertically from the surface of the Ti foil.

Figure S2 shows the SEM images of the top surface of the TiO_2 nanotube arrays with Ag nanofilm after heat treatment at different temperatures. Without any teat treatment, the Ag nanofilm generally covered the top of the TiO_2 nanotube arrays (Figure S2a) and made the topology of nanotubes indistinguishable. With the heat treatment at 300 °C for 2 h, dewetting of the Ag nanofilm and the migration/diffusion of Ag atoms into the TiO_2 nanotube arrays

occurred. The Ag nanofilm became irregular, and the amount of Ag on the top of the TiO_2 nanotube arrays decreased. The irregularity of the Ag nanofilm increases with the increase of the heat-treatment temperature for the same heating time, and the amount of Ag on the top of the TiO_2 nanotube arrays decreases with the increase of the heating time and heat-treatment temperature.

Figure S3 shows the line scan of EDS of the cross section of the TiO_2 nanotube arrays with Ag nanofilm, which was heat-treated at 400 °C for 2 h. The line scan reveals the migration/diffusion of Ag into the TiO_2 nanotube arrays.

Figure S4 shows the EDX patterns of the TiO_2 nanotube arrays with Ag nanofilm after heat treatment at 500 °C for 2 h. The EDX result reveals the presence of Ti, O, and Ag, confirming the existence of Ag around the TiO_2 nanotubes.

Figure S5 shows the TEM images of the TiO_2 nanotube arrays with Ag nanofilm after heat treatment at 500 °C for 1 h. The images reveal the presence of Ag nanoparticles on the surface of TiO_2 nanotubes and the characteristic lattice fringe of 3.52 Å for TiO_2 nanotubes and 2.36 Å for Ag nanoparticles.

Figure S6 shows the SEM images of anatase TiO_2 nanotube arrays with Ag nanofilm after heat treatment at 500 °C for 2 h. The images reveal the presence of Ag on the outmost surface of TiO_2 nanotubes, suggesting that Ag atoms diffused through the space between anatase TiO_2 nanotubes, similar to the amorphous TiO_2 nanotubes.

Figure S7 shows the TEM images of the Ag/TiO_2 nanotubes after heat treatment at 500 °C for 2 h and the corresponding size distribution of Ag nanocrystals.

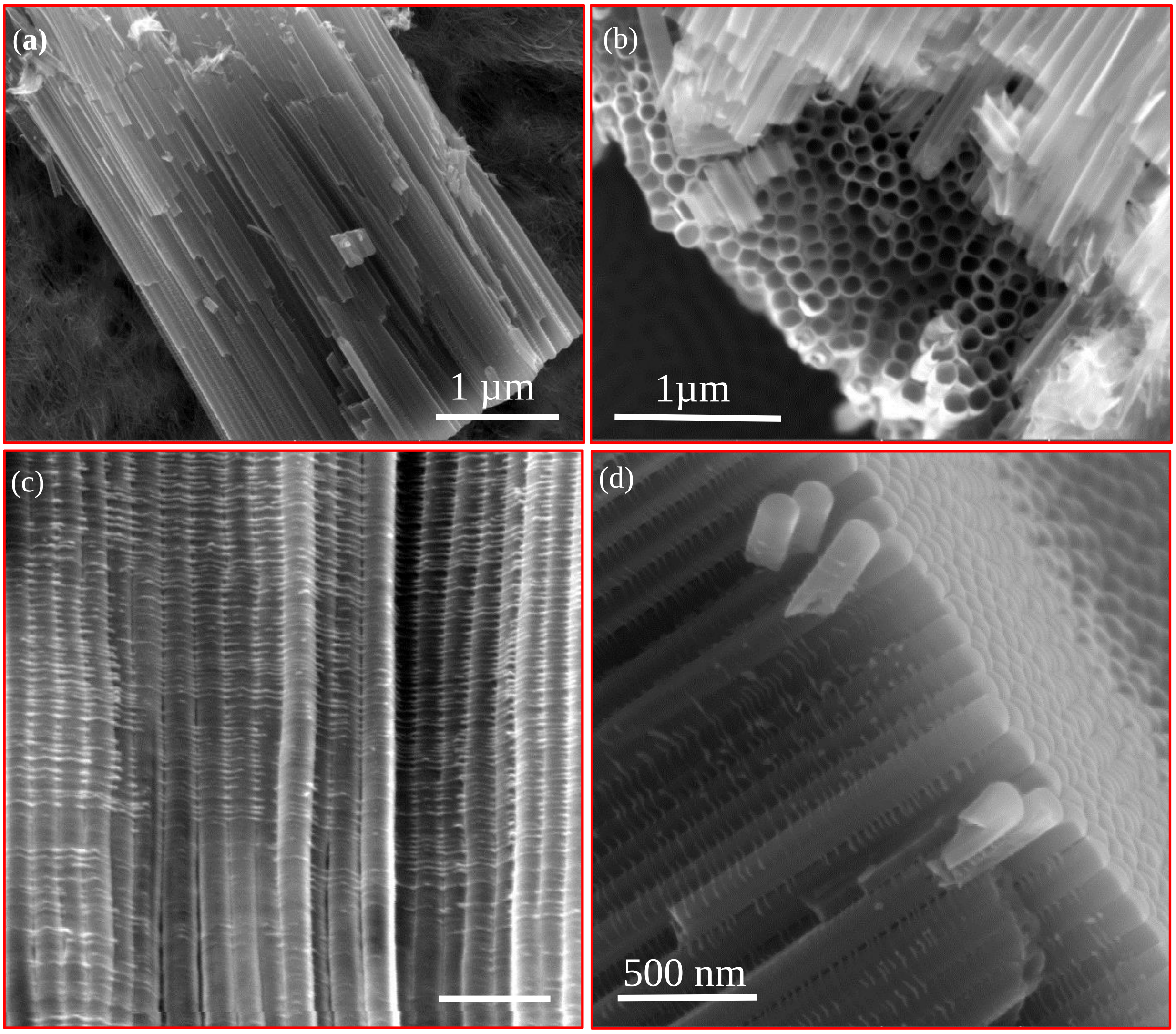

Figure S1: SEM images of pure TiO_2 nanotube arrays prepared by one-step anodization; (a) overview of the TiO_2 nanotube arrays, (b) top topology of the TiO_2 nanotube arrays, (c) side view of the TiO_2 nanotube arrays showing the bamboo-like structures on the surface of the TiO_2 nanotube arrays, and (d) bottom surface of the TiO_2 nanotube arrays.

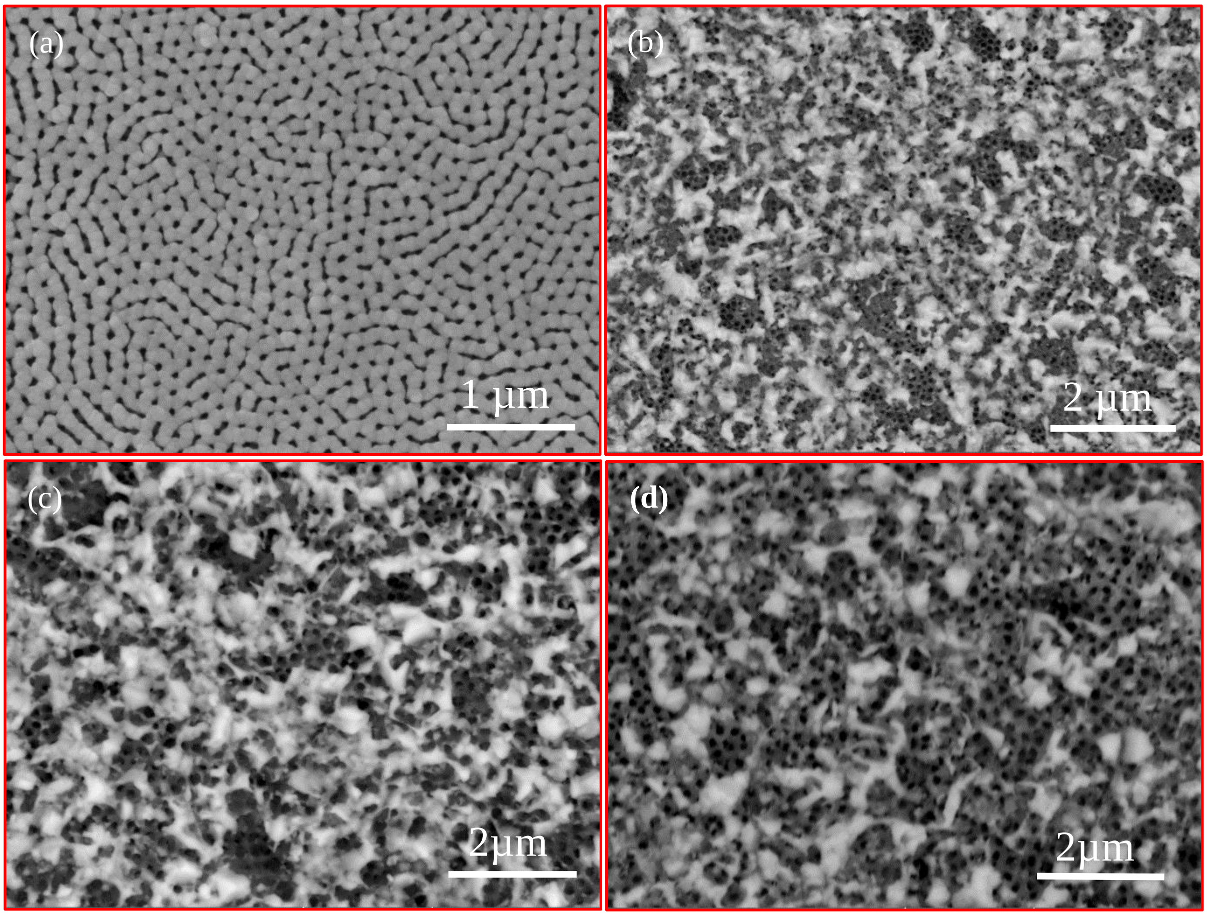

Figure S2: SEM images of the top surface of the TiO_2 nanotube arrays with Ag nanofilm after heat treatment at different temperatures; (a) before heat treatment; (b) 300 °C for 2 h; (c) 400 °C for 2 h; (d) 500 °C for 2 h.

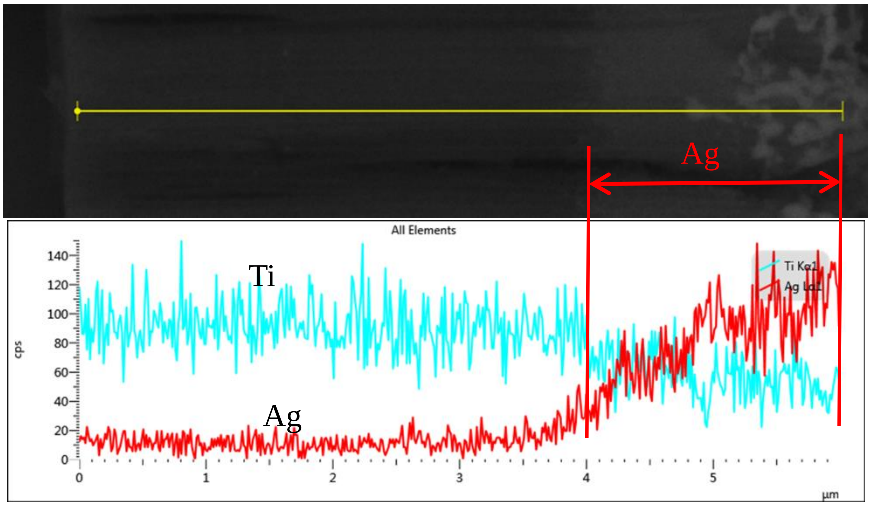

Figure S3: EDS line scan of the TiO_2 nanotube arrays with Ag nanofilm after the heat treatment at 400 °C for 2 h, showing the migration/diffusion of Ag into the TiO_2 nanotube arrays.

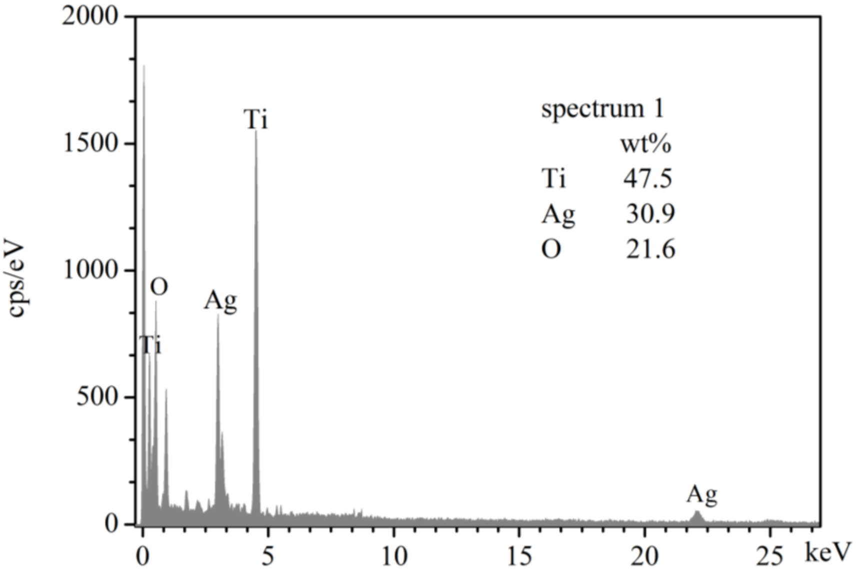

Figure S4: EDX pattern of the TiO_2 nanotube arrays with Ag nanofilm after heat treatment at 500 °C for 2 h.

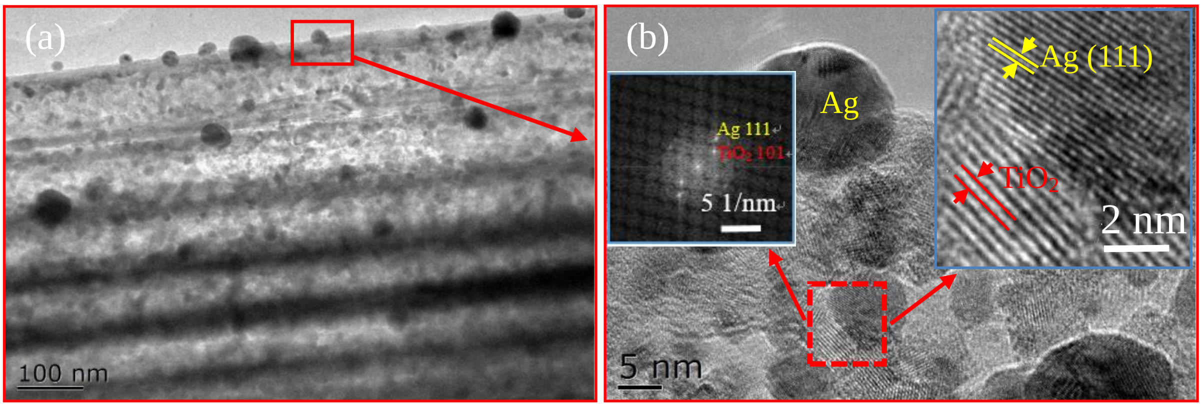

Figure S5: TEM images of the TiO_2 nanotube arrays with Ag nanofilm after heat treatment at 500 °C for 1 h; (a) Ag nanoparticles on the surface of TiO_2 nanotubes, and (b) HRTEM image showing the characteristic lattice fringes of 3.52 Å for TiO_2 nanotubes and 2.36 Å for Ag.

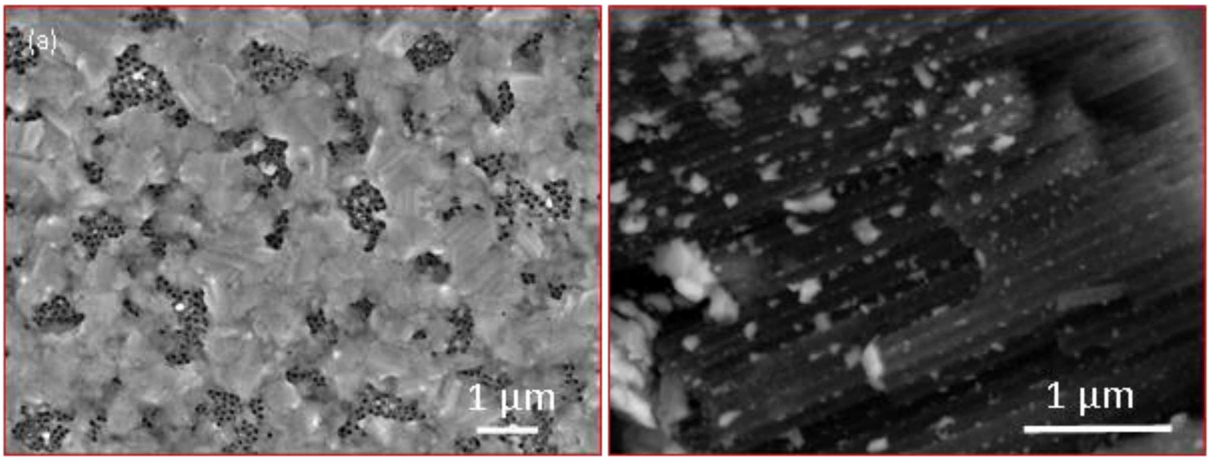

Figure S6: SEM images of anatase TiO_2 nanotube arrays with Ag nanofilm after heat treatment at 500 °C for 2 h; (a) Top view, and (b) side view showing the presence of Ag on the outmost surface of TiO_2 nanotubes.

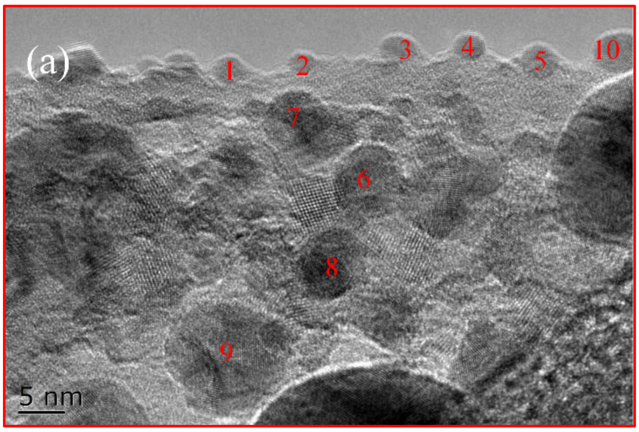

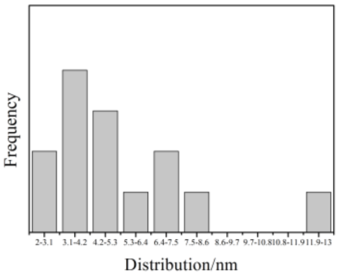

total	10
Max./nm	12.78
Min./nm	2.55
Mean/nm	5.55

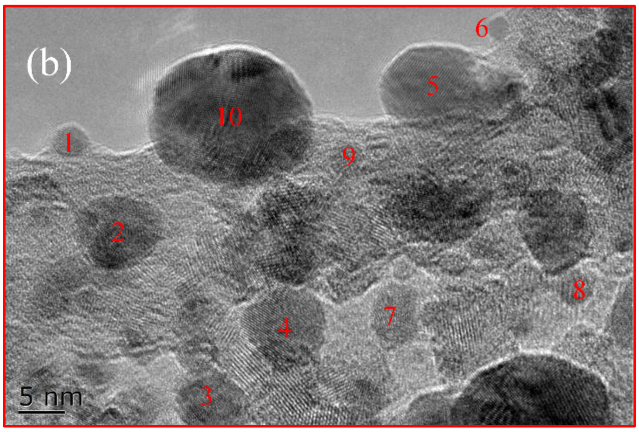

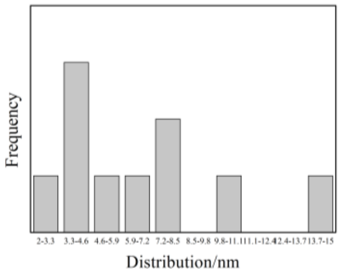

total	10
Max./nm	14.54
Min./nm	2.62
Mean/nm	6.68

Figure S7: TEM images of the Ag/TiO_2 nanotubes after heat treatment at 500 °C for 2 h and the corresponding size distribution of Ag nanocrystals.
